# Complete mitochondrial genome of *Haematopus ostralegus* (Charadriiformes: Haematopodidae)

**DOI:** 10.1080/23802359.2017.1292474

**Published:** 2017-02-23

**Authors:** Mu-Yeong Lee, Hey Sook Jeon, Yu-Seong Choi, Sungbae Joo, Junghwa An

**Affiliations:** a Animal Resources Division, National Institute of Biological Resources, Incheon, Republic of Korea;; b Division of Basic Research, National Institute of Ecology, Seocheon, Chungcheongnam-do, Republic of Korea

**Keywords:** *Haematopus ostralegus*, genome, mitochondrion, Eurasian oystercatcher

## Abstract

The Eurasian oystercatcher (*Haematopus ostralegus*), Near Threatened in the IUCN red list, was designated classified endangered species II by the Ministry of Environment of Korea and a Natural Monument (No. 326) by the Cultural Heritage Administration of Korea. In this study, the complete mitochondrial genome (16,798 bp) of *H. ostralegus* was determined for the first time, including 13 protein-coding genes, 2 ribosomal RNA genes, 22 transfer RNA genes, and 1 non-coding region. The overall base composition was A (31.4%), C (31.3%), G (13.8%), and T (23.5%), so the percentage of A and T (54.9%) was slightly higher than that of G and C. A phylogenetic analysis using concatenated mitogenomes revealed that the family Haematopodidae has a closer relationship with the family Recurvirostridae and *H. ostralegus* and *H. ater* clustered together. The results are expected to provide useful resources for species identification and further phylogenetic studies of genus *Haematopus*.

The Eurasian oystercatcher, *Haematopus ostralegus* (Charadriiformes: Haematopodidae), is one of the largest waders with wide distribution. The population size has been decreasing because of overexploitation of its prey and habitat degradation due to to rapid and extensive land reclamation and climate change (BirdLife International 2016). This species, Near Threatened (NT) on the IUCN red list, was classified endangered species II by the Ministry of Environment of Korea and designated a Natural Monument (No. 326) by the Cultural Heritage Administration of Korea. There are three subspecies of *H. ostralegus* (*ostralegus* in Europe, *longipes* from central Asia and Russia, and *osculans* from East Asia). South Korea plays an important role of wintering grounds for *H. o. osculans*. About 50% of the population wintered in South Korea (Melville et al. 2014). Their mitochondrial genome was not determined in spite of the urgent need for assessing the taxonomic status of *H. o. osculans* (Del Hoyo et al. 1996; Chandler 2009; Livezey 2010).

The specimen (IN716) of *H*. *ostralegus* was collected from Seocheon-gun, Chungcheongnam-do, South Korea, after obtaining the permission from the related regulation (from the Cultural Heritage Administration of Korea). It was deposited in the National Institute of Biological Resources at Incheon, South Korea. Total genomic DNA was isolated from a blood sample using DNeasy Blood & Tissue Kit (Qiagen, Valencia, CA) according to the manufacture’s instruction. The mitochondrial genome of *H*. *ostralegus* was amplified and sequenced by 16 pairs of primers. Consensus sequences were assembled and proofread by eyes in Geneious Pro v8.1.9 (Biomatters; Kearse et al. 2012), which was deposited into GenBank with the accession number (KY419886). The circular mitogenome of *H*. *ostralegus* was 16,798 bp in length and contains 13 protein-coding genes, 22 transfer RNA genes, 2 ribosomal RNA genes, and 1 control region (D-loop region). The mitogenome had tandem repeats of 7 bp repeat units and AACAAAC tandem repeats in D-loop region (1249 bp). The overall base composition of the mitogenome was 31.4%, 31.3%, 13.8%, and 23.5% for A, C, G, and T, respectively. So, the percentage of A and T (54.9%) was slightly biased. As similar to the typical vertebrate mitogenomes, all the genes in *H. ostralegus* were distributed on the H-strand, except for the *ND6* subunit gene and eight tRNAs, which were encoded on the L-stand. Interestingly, the *ND3* gene had a single extra base ‘C’ at position 174, which was reported in some birds and turtles (Mindell et al. 1998). This phenomenon was also found in *H. ater* (AY074886).

Sequences of 13 protein-coding genes (11,396 bp) were concatenated to understand the phylogenetic relationships with neighbour-joining (NJ) methods by MEGA 6 (Tamura et al. 2013). The phylogenetic tree (Figure 1) indicated that the family Haematopodidae has a closer relationship with the family Recurvirostridae and *H. ostralegus* and *H. ater* clustered together. The results of this study are expected to contribute the improvement of understanding of phylogenetic relationships of genus Haematopus species and provide useful data for molecular species identification.

**Figure 1.**
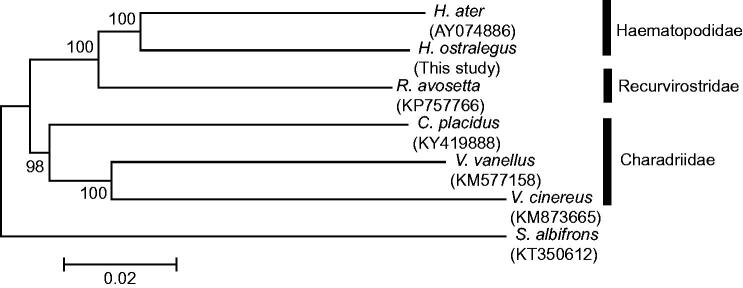
A phylogenetic tree of six species of family Haematopodidae, Recurvirostridae, and Charadriidae based on the concatenated nucleotide sequences of 13 protein-coding genes. Bootstrap replicates were performed 1000 times. Numbers on nodes indicate the bootstrap value.
